# P03-06 Longitudinal associations among cardiorespiratory fitness and objectively measured moderate-to-vigorous physical activity of Finnish schoolchildren

**DOI:** 10.1093/eurpub/ckac095.042

**Published:** 2022-08-29

**Authors:** Timo Jaakkola, Mikko Huhtiniemi, Kasper Salin

**Affiliations:** Faculty of Sport and Health Sciences, University of Jyväskylä, Jyväskylä, Finland

**Keywords:** Physicl activity, cardiorespiratory fitness, regression analysis, youth

## Abstract

**Background:**

Children and adolescents' engagement in physical activity has decreased in most Western countries across the previous three decades. Therefore, increasing number of researchers are investigating antecedents of physical activity engagement in childhood and adolescence. This longitudinal study investigated if cardiorespiratory fitness measured at Grade 5 explained objectively measured moderate-to-vigorous physical activity (MVPA) at grade 7.

**Methods:**

The sample of the study included 169 (62 boys and 107 girls, Mage = 11.27, *SD* = .32 at the beginning of the study) Finnish PE students. Cardiorespiratory fitness was measured by 20 meters shuttle run test and MVPA by Actigraph wGT3X+ accelerometers. Sex, BMI and MVPA score at Grade 5 were used as covariates in the analysis. Hierarchical multiple regression analysis was conducted as follows: (a) sex, BMI and MVPA score at Grade 5 were set as covariates on the first step of the analysis; (b) cardiorespiratory fitness measured at Grade 5 was set as independent variable on the second step of the analysis; c) MVPA measured at Grade 7 was set as dependent variable on the third step of the analysis.

**Results:**

Regression analysis demonstrated that sex, BMI and MVPA at Grade 5 were significant variables (R2 = 0.235; F = 16.925; p = 0.000) predicting variance in MVPA score at Grade 7. More specifically, results indicated that BMI (β = 0.161; P < 0.026) and MVPA at Grade 5 (β = 0.397; P < 0.000) were significant predictors of MVPA score at Grade 7, whereas sex (β = 0.023; P < 0.738) was not. Subsequently, cardiorespiratory fitness measured at Grade 5 improved predictive strength of the model for MVPA at Grade 7 (R2 = 0.284; R2 change = 0.049; F = 16.250; P < 0.001). Results showed that cardiorespiratory fitness at Grade 5 (β = 0.264, P < 0.001) was a significant predictor of MVPA at Grade 7.

**Conclusions:**

Results of this study demonstrated that cardiorespiratory fitness is an important antecedent of MVPA from childhood to adolescence. However, the portion cardiorespiratory fitness predicted MVPA was relatively low. Intensive physical activities within elementary school years are strongly encouraged.

